# A scale to measure the perceived quality of mHealth by elderly patients with hypertension in China

**DOI:** 10.1186/s12913-023-09357-z

**Published:** 2023-04-10

**Authors:** Minjiang Guo, Lanting Lyu

**Affiliations:** 1 Institute of Medical Information, Chinese Academy of Medical Sciences, 3 Yabao Road, Chaoyang District, Beijing, 100020 China; 2 School of Public Administration and Policy, Renmin University of China, Beijing, 100872 China; 3 Health Technology Assessment and Health Policy Research Group at Renmin, University of China (RUCTAG), Beijing, 100872 China

**Keywords:** Hypertension management, Quality evaluation, Quality perception, Mobile health, Aging population

## Abstract

**Background:**

The elderly population in China is growing, with hypertension being the most prevalent chronic disease in older adults. Despite the rapid penetration and efficient management effect of mobile health on hypertension healthcare, elderly patients are often less adopted and continue to use mobile health services. Quality perception significantly affects an individual’s satisfaction and continued intention to use mobile health services. The evaluation of the significant factors affecting mobile health quality perception by elderly individuals remains largely unexplored. The aim of this study was to develop and validate an evaluation scale to measure the perceived quality of mobile health applications for hypertension and determine the underlying influencing factors.

**Methods:**

A cross-sectional survey was conducted between November 2018 and October 2019. A quality evaluation scale with three factors and seven indicators was developed based on the Information Systems Success model. Data was analyzed using structural equations modelling (SEM) and one-way analysis of variance (ANOVA). All tests were two-sided and statistically significant at *P* < 0.05.

**Results:**

The proposed mobile health application quality evaluation scale from the perspective of the elderly was shown to be a hierarchical, multidimensional construct with valid reliability, convergent validity and discriminant validity, which consists of three factors and seven indicators. The SEM results suggested that information quality and service quality had a significant impact on the satisfaction of elderly individual’s with mobile health applications for hypertension management. The results also suggest that the elderly individuals had a low evaluation of mobile medical service quality(4.06 ± 0.70), while the score of information quality was the highest, with an average score of 4.36(SD 0.83) out of 5. Male patients were shown to more readily accept mobile health applications, with their perception of system quality being 0.27 higher than female ones (95% CI 0.00 ~ 0.52; *P* < .05). Patients with 1–5 years hypertension histories assessed the system (95% CI 0.03 ~ 0.63; *P* < .05), information (95% CI 0.11 ~ 0.65; *P* < .05), and service quality (95% CI 0.00 ~ 0.47; *P* < .05) higher than those with hypertension histories > 10 years. Elderly patients who regularly visited primary hospitals assessed the information quality 0.13 higher (95% CI -0.08 ~ 0.34; *P* < .05) than those visited tertiary hospitals.

**Conclusions:**

These findings have significant implications for theoretical and practical research on mobile health application quality evaluation, which will be helpful for policymakers and mobile health providers in improving the context and utilisation of mobile health to include elderly users. More mobile health applications attributes, such as timely information and interactive services that meet the characteristics of elderly patients with different mental and health demands need to be considered. Deeply embedding mobile health into primary health services is recommended to help increase the perceived quality of mobile health, and ensure the continuous use.

**Supplementary Information:**

The online version contains supplementary material available at 10.1186/s12913-023-09357-z.

## Introduction

The spectrum of causes of death has changed significantly worldwide from infectious to chronic non-infectious diseases (NCD), such as cardiovascular and cerebrovascular diseases [1]. According to the Report on Cardiovascular Diseases in China states that cardiovascular deaths account for the top cause of death among Chinese residents, and hypertension is the most prevalent cardiovascular disease [2]. Older age is significantly associated with an increased risk of hypertension. Based on the 2012–2015 China Hypertension Survey, the prevalence rate of people aged over 65 had exceeded 50%, but the control rate was only 18.4% for people aged 65–74 years and 17.0% for those aged over 75 years, which had led to a great burden on older patients with hypertension and their families [3]. According to the seventh national census of China, the number of people over 65 years has reached 190 million, accounting for 13.5% of the total population [4]. Effective management and control of hypertension among older adults has significant meaning in the realization of the ‘Health China’ strategy.

Mobile health (mHealth) is the use of mobile technologies to improve health care processes and outcomes [5]. The popularization of smartphones has improved access to mHealth uptake. More than 2000 mHealth applications(apps) were founded in China by 2016, with 81,604 hospital-based specialists and 400,344 patients registering on these platforms [6]. It is also widely used in other developing and developed countries as well. Between 1999 and 2016, approximately 26 mHealth initiatives were introduced in Bangladesh [7]. The number of health-related apps exceeded 318,000 in the United States [8]. According to statistical data from the China Internet Network Information Center (CNNIC), the outbreak of the COVID-19 pandemic has caused a sharp rise in mHealth utilization [9]. It has proven effective in facilitating primary healthcare service delivery and enhancing the service productivity for patients with chronic disease through the characteristics of timely mutual communication from anywhere [10].

The promising effect of mHealth on health management effect relies heavily on users’ willingness to accept and continue using it. However, as time passed, the continued use rate of mHealth had declined rapidly. Kim, K.-H [11] found that after its first run of usage, 45% of patients did not consistently use mHealth services. According to a study in the USA, the retention rate of mHealth apps was less than 50% after 30-days of usage [12]. On the other hand, older adults who are more susceptible to chronic diseases, experience difficulty using mHealth promising. A survey from the Pew Research Center in 2012 showed that 31% of mobile phone users utilized mHealth for their health problems via their smartphones, but the adoption and utilization rate of mHealth services was low among users aged > 65 years [13]. In a survey on the cognition and use of medical care through the internet in China, the adoption rate of internet medical care among the elderly over 60 years old was only 15.2%, much lower than the 92.8% among the population under 60 years [14]. This disparity in utilization indicates that mHealth enterprises should pay more attention to improving the perception of the elderly population toward their products. Researches in the information system field has shed light on strong influence of quality perceptions on one’s satisfaction and intention to continue using mHealth. Nevertheless, the definition of quality in this context remains ambiguous, while related indicators are scattered under factors such as ‘perceived usefulness’ and ‘facilitating conditions’. It is crucial to systematically analyze the quality factors based on patient concerns to elicit the mHealth participation mechanism of elderly people, which could help prepare guidelines for mHealth development.

The perceived quality of mHealth apps is a multidimensional concept, and because of its service-oriented nature, the evaluation should be context-dependent. Although many studies have probed this aspect of mHealth services, few have focused on specific diseases. Given the reality that hypertension has become the predominant death risk factor worldwide and intensifies with aging, there is a need to develop an reliable instrument supporting the evaluation of all dimensions of quality in order to fulfil the potential of mHealth in hypertension management.

The main aim of this study was to develop and validate a multidimensional, hierarchical quality scale for measuring mHealth quality from the perspective of elderly patients with hypertension. To complete this, we divided our research into three parts. First, we determined the nature and dimensions related to our research context. Subsequently, a quality evaluation scale from the perspective of elderly hypertensive patients was developed based on the Information Systems Success Model. Finally, a survey of patients with hypertension aged > 65 in China was conducted to examine the reliability and validity of the scale, and the relationship between the quality factors and the satisfaction was tested. Overall, this study answers the following two research questions:

- 1. What are the significant factors affecting mHealth quality perception by elderly individuals with hypertension in China?
- 2. Does these quality factors impact patients’ satisfaction with mHealth apps?

### Literature review

Quality has been recognized as one of the most important determinants of the long-term success of electronic services in electronic markets. The combination of information system theory and behavior theory is widely applied in the investigation of mHealth and their usage behavior. Expectation confirmation model (ECM) [15], Information systems success model (ISS) [16], Technology Acceptance Model(TAM), and Unified Theory of Acceptance and Use of Technology(UTAUT) [17] are four extensively used theories in this research field, and their applicability has been well recognized with mixed statistical significance, direction, and magnitude [18]. Multiple constructs derived from these theories are closely related and confirmed by researches and can be divided into three categories. One category reflects application’s characteristics, such as system, information and service quality, and is derived from ISS. Nisha et al. [19] proposed a conceptual model to examine the factors primarily related to system and information quality to understand users’ intentions for using mHealth services in Bangladesh. Peiyu et al. [20] built a mHealth application quality evaluation system that included four primary indicators: usability, security, information content and technicality, these indicators focused on the technology attributes of mHealth. Increasing research focusing on mHealth use has highlighted the need for investigating factors influencing the adoption of information systems from a human-centric perspective. In 2003, DeLone and McLean improved the original information system success model by introducing service quality to the model, and paralleling it with system and information Quality, which jointly influences system use and user satisfaction [21]. This has led to increase attention to the influence of service quality on mHealth quality and usage behavior. Palas et al. [22] showed that service quality had significant effects on the use intention and behavior of mHealth services by elderly individuals. Akter et al. [23] innovatively proposed an instrument to measure the user perceived service quality of mHealth apps, and they defined it as a consumer’s judgment of or impression about an mHealth platform’s overall excellence or superiority, this definition also included the system and information quality related to the service. Another category often considered is the user’s feelings about the experience of using an mHealth app. Perceived usefulness, perceived ease of use, expectation confirmation, performance expectation, and other constructs following behavior theories have been proven to have strong explanatory power for mHealth use behavior [24], and these are usually affected by the app’s characteristics. Li et al. [25] included the information and functional features of HIV mobile follow-up apps as product factors in their research on the influencing factors of behavioral intention, and showed that these factors indirectly promoted users' intentions to utilize the apps by influencing perceived usefulness, ease of use and innovation. The third category of influencing constructs includes the social and economic environment of users, such as subjected norms, social influence, and facilitating conditions. These factors influence the motivation of individuals to use mHealth apps by shaping the objective environment that promotes the development of mHealth, this is the external cause that promotes its use, thereby affecting use behavior. Undoubtedly, the perceived quality of mHealth apps are a fundamental factor that influence feelings and utilization behaviors. Perceptions of poor quality of care may discourage patients from using available service systems [26]. The evaluation of quality perception does not only identify problems that lead to users not being likely to adopt and continue to use mHealth apps, but also provide developers with relevant information on how to optimize the quality of the product. However, few studies have investigated elderly individuals’ perception of mHealth apps for specific chronic diseases. The main population with chronic diseases, is the elderly population; this group has several barriers in using mHealth apps, such as the general problem of continuous use, motivation, perception, and the decline in cognition and physical abilities [27]. Therefore, it is important to evaluate mHealth quality from the perspective of elderly individuals; this will be of great value in expanding the application of mHealth and improving the flexibility and sustainability of chronic disease management in the elderly population.

### Theoretical framework

DeLone and McLean Model of Information Systems Success(ISS) [21] delivered a comprehensive, multidimensional framework for analysing the quality of different aspects of information products and has been proven suitable for analysing the quality of health information systems [28], especially their adoption and post adoption mechanisms in the electronic health record system, emergency response medical information system [29],and telehealth [30].This study adopted ISS as the mainly theoretical backbone and selected indicators from previous research. Three primary dimensions were to represent the quality perception: system, information and service quality. System quality is defined as “desirable characteristics of an IS” [31] which refers to the user’s perceptions regarding the technical level of mHealth apps, whereas ease of use [32], functionality [30], system efficiency, or flexibility [33] are commonly referred indicators. For hypertension management, mHealth apps often require multiple functions such as recording, reminding, communication, and consultation to meet the needs of different types of patients, and functional insufficiency has become an important factor affecting app quality [34, 35]. Simultaneously, owing to the characteristics of continuous care for chronic diseases, health management apps need to have good compatibility with various devices. Consequently, four indicators were selected to represent the perception of the system quality of hypertension management apps: ease of use, ease of navigation, functionality and inter-device compatibility. Information quality is defined as the degree to which a person believes that using a particular system will enhance their job performance [36]. For hypertension management, an mHealth app needs not only expert knowledge for popularization, propaganda and education, but also advice and feedback from physicians to instruct patients on hypertension management at home. To assess how effectively mHealth apps assist patients in hypertension management, accuracy, relevance, and timeliness are the most important criteria for mHealth app users [37, 38], which were selected as mainly indicators in our research for the construct of information quality. Service quality is a newly added construct in ISS model which has been increasingly recognized as a crucial influencing factor for information products success. As for hypertension management, the service quality is reflected not only in the matching between the characteristics of apps and the needs of patients, but also in the coordination of services and affordability. Communication interactivity [39], service customization [40] and affordability [41] are the three primary evaluation indicators for chronic disease management. Finally, according to ISS model, all the three factors are supposed to affect the user’s degree of satisfaction with mHealth apps.

**Fig. 1:**
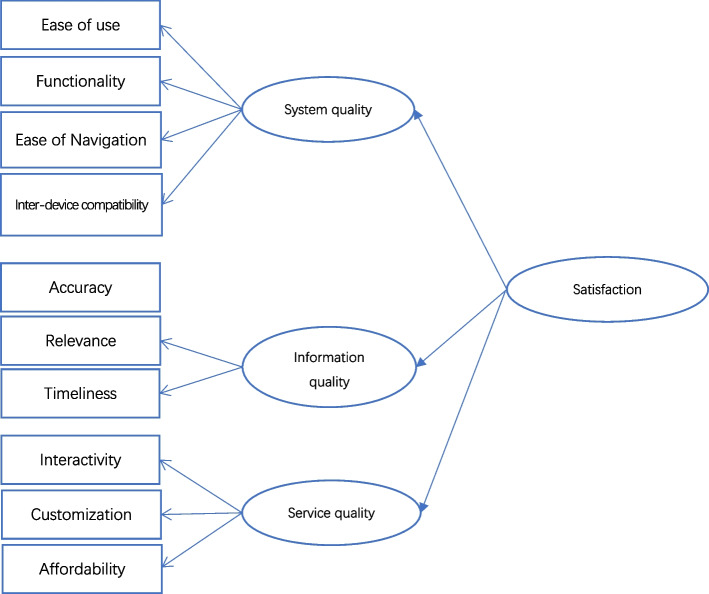
Theoretical framework

This study aims to establish a quality-evaluation theoretical framework that contains three factors and the indicators (Fig. 1). To verify the impact paths of the quality factors on the degree of user satisfaction, the following three hypotheses were developed: H1: system quality has a positive impact on the degree of satisfaction with mHealth apps experienced by elderly individuals; H2: information quality has a positive impact on the degree of satisfaction with mHealth apps experienced by elderly individuals; H3: service quality has a positive impact on the degree of satisfaction with mHealth apps experienced by elderly individuals.

### Study population and methods

#### Questionnaire design and data collection

The measurement items for the factors within the framework were derived from the previous studies and revised based on mHealth services for hypertension management in China to confirm the validity and reliability of the theoretical framework. The final items derived from the framework are presented in Appendix A. An open web-based survey was designed following the Checklist for Reporting Results of Internet E-Surveys (CHERRIES) checklist [42]. The web-based survey was developed using an online survey tool, Survey Stars, which generated quick response (QR) codes and hyperlinks to the questionnaire for distribution. The content of the questionnaire was consisted of three parts: (1) demographic characteristics, including gender, age, and education; (2) hypertension and treatment history, including the duration of disease, treatment regularity, regular treatment frequency and regular hospital-level of treatment; and (3) the hypertension management apps typically used by the participants and their perceived quality evaluation of the items developed from the theoretical framework. Four questions on system quality perception, three on information quality perception, and three on service quality perception were included. A standard five-point Likert scale, ranging from strongly agree (5) to strongly disagree (1), was used to generate the cognitive data. The content of the questionnaire was approved by a group of experts.

The questionnaire was allocated to several online communities of patients with hypertension, to capture a representative sample population of elderly individuals who use hypertension management apps in China. Patients who participated in the survey also shared the questionnaire hyperlink with their friends who suffer from hypertension. The questionnaire was administered from November 2018 to October 2019, and 358 patients participated in the survey. After excluding 120 individuals who were < 65 years-of-age, 238 valid questionnaires were received.

### Scale validity and reliability

The scale proposed for this research was analyzed through structural equation modelling (SEM) technique which has been recognized as a favorable technique for estimating hierarchical models with moderating and mediating effects [43]. Scale testing followed a two-step approach. First, a measurement model was assessed using confirmatory factor analysis (CFA). Then, the causal association between the independent and dependent variables was tested with SEM. As recommended by previous researches, Cronbach’s Alpha and composite reliability (CR) were used to evaluate the internal reliability with a value of 0.7 as the acceptable level according to previous researches [44]. The square root of Average variance extracted(AVE) was applied to assess the scale’s discriminant validity, while the calculated square root of the AVE of one factor should be greater than the corresponding correlation between the factor and the other factors according to Md Abdul Kaium [45]. Normed chi-square (${X}_{2}/df$), root mean square error of approximation (RMSEA), tucker-Lewis index (TLI), and the comparative fit index (CFI) were chose to evaluate the overall fit of the research model. As an acceptable scale, ${X}_{2}/df$ value was suggested not exceeding 3.00. RMSEA value as a measure of absolute fit should be lower than 0.08. TLI and CFI values determined the model’s incremental fit need to be higher than 0.90 [46].

### Data analysis

Data analyses were performed using IBM SPSS statistical software (version 22.0; IBM Corp). Categorical variables, such as education, were described using frequency counts and expressed as n(%), and normally distributed continuous variables were described as mean ± standard deviation. In addition to examining the quality perception evaluation model, one-way analysis of variance (ANOVA) was conducted to investigate the relationship between the characteristics of the surveyed patients and their evaluation results. All analyses were two-tailed, and statistical significance was set at *P* < 0.05.

### Ethical consent

Ethical approval was obtained from the ethics committee of IMICAMS (reference number IMICAMS/02/21/HREC). All methods were performed in accordance with relevant guidelines and regulations. Patients were informed of the purpose and confidentiality of the survey. A statement of consent was included with a brief description of the study in the introduction of the questionnaire, and the consent of the participants was implied by the completion and return of the questionnaire, which was confidential and anonymous.

## Results

### Sample characteristics

The demographic, hypertension, and treatment profiles are listed in Table 1. Of the 238 patients, 71.8% were male and 79.8% had a junior college level education or below. A total of 133 patients (55.9%) had hypertension for > 10 years. In terms of treatment history, 166 surveyed patients (69.7%) had a history of regular hospital visits. Among them, 102 patients (61.4%) chose tertiary hospitals as their regular follow-up facilities. Of these patients, 30.7% visit the hospital every month, while 36.2% percent only visit the hospital every year.

**Table 1:** The demographic and hypertension profiles of respondents

Characteristic	Number of People (persons)	Proportion (%)
**Gender**		
Male	171	71.8%
Female	67	28.2%
**Education Level**		
Junior college and below	190	79.8%
Undergraduate and above	48	20.2%
**Region**		
Provinces in East China	109	45.8%
Provinces in Central China	85	35.7%
Provinces in West China	44	18.5%
**Hypertension History**		
1–5 years	49	20.6%
6–10 years	56	23.5%
Over 10 years	133	55.9%
**Regular Visit**		
Yes	166	69.7%
No	72	30.3%
**Regularly Visit Hospital Level (among regular visit patients)**		
Primary	38	22.9%
Secondary	26	15.7%
Tertiary	102	61.4%
**Regular Visit Frequency (among regular visit patients)**		
Every month	51	30.7%
Every 3 months	55	33.1%
Every year	60	36.2%

### Measurement model

 The CFA result was presents in Table 2. The values of Cronbach’s alpha and CR were above 0.875 and 0.880, respectively. AVE values ranged from 0.678–0.721. All the measurements exceeded the recommended level, which implies that all factors in the measurement model had adequate internal reliability and convergent validity.

**Table 2:** The measurement model

Dimensions	Cronbach’s alpha	CR	AVE
System quality	0.894	0.894	0.678
Information quality	0.885	0.886	0.721
Service quality	0.875	0.880	0.710

*CR* Composite reliability, *AVE* Average variance extracted

The original square root of the AVE of ‘system quality’ and ‘information quality’ was slightly less than the maximum value of the correlation coefficient value. Therefore, three indicators with lower standard factor loading values were removed; these factors were ‘compatibility’, ‘ease of use’ and ‘accuracy’. After the modification, the square root of the AVE of all the three factors satisfied the measurement standard (Table 3). Therefore, the reliability, convergent validity and discriminant validity of the modified evaluation model have been confirmed.

**Table 3:** Correlation matrix and the square root of the AVE

Factors	system quality	information quality	service quality
System quality	0.884		
Information quality	0.817	0.855	
Service quality	0.754	0.791	0.842

### Structural model

The structural model was used to estimate the significance of the impact paths from the hypotheses. The results of these indices are listed in Table 4, along with their good fit standards recommended to be acceptable.

**Table 4:** Fitting index of the research model

The goodness of fit indices	Cut-off value	Result	Status
${X}_{2}/df$	< 3.00	1.702	Acceptable
RMSEA	< 0.10	0.054	Acceptable
TLI	> 0.90	0.988	Acceptable
CFI	> 0.90	0.994	Acceptable

${\mathrm{X}}_{2}/\mathrm{df}$, Chi-squared divided by degrees of freedom, *RMSEA* Root mean square error of approximation, *TLI* Tucker-Lewis index, *CFI* Comparative fit index

**Fig. 2:**
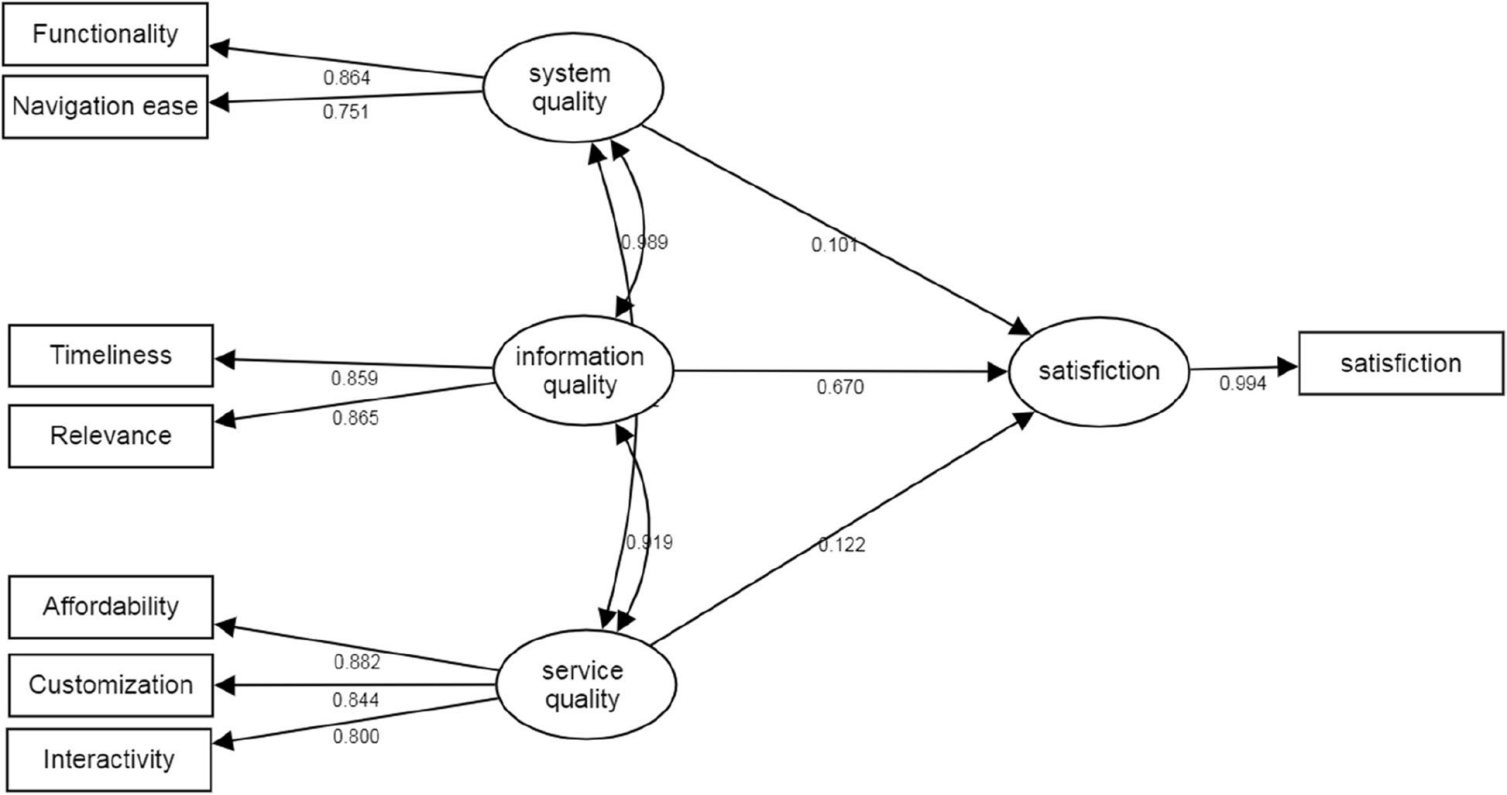
Results of the structural model with loading values

The loading values of the three factors for the indicators displayed in Fig. 2 that ‘system quality’, ‘information quality’, ‘service quality’ can be measured through combinations of these indicators. The relationship between the variables in the proposed model was evaluated using path coefficients at a significant level of 0.05 (Table 5). The results indicated that ‘information quality’ and ‘service quality’ all significantly affect the user’s degree of satisfaction with mHealth apps.

**Table 5:** Results of the path coefficients from the hypothesis

Paths			Path coefficient	Standard error	Z value	*P* value	Standardized path coefficient
System quality	→	Satisfaction	0.102	0.234	0.782	0.434	0.101
Information quality	→	Satisfaction	0.741	0.233	2.926	0.003	0.670^a^
Service quality	→	Satisfaction	0.127	0.093	2.142	0.032	0.122^a^

^a^ *P* < .05

### Quality analysis

The quality scores from the survey population were analyzed. As for the three factors, information had the highest mean quality (4.36 ± 0.83), followed by system (4.20 ± 0.91), and service (4.06 ± 0.70) quality. To explore the influencing factors, the quality scores in different patient’s groups were analyzed and a one-way ANOVA was conducted to evaluate app quality evaluation in regards to different patients feature. Elderly male patients were generally more satisfied with the quality of hypertension management apps than female patients, especially with regards to system quality (0.27, 95%CI:-0.00 ~ 0.52; *P* < 0.05). Elderly patients with ‘1–5 years hypertension history’ rated system (0.33, 95%CI: 0.03 ~ 0.63; *P* < 0.05), information (0.38, 95%CI: 0.11 ~ 0.65, *P* < 0.05) and service (0.24, 95%CI: 0.00 ~ 0.47, *P* < 0.05) quality significantly higher than patients with more than 10 years hypertension history. Patients who had regular hospital visits every three months evaluated the service quality significantly higher than those who visited the hospital every month (0.29, 95%CI: 0.02 ~ 0.55, *P* < 0.05). The quality scores in different patient groups and the multivariate linear regression analysis are summarized in Tables 6 and 7.

**Table 6:** MHealth app quality scores in different elderly patient’s groups

Characteristic	System Quality	Information quality	Service quality
**Gender**			
Male	4.39	4.46	4.11
Female	4.12	4.32	4.04
**Education Level**			
Junior college and below	4.26	4.41	4.09
Undergraduate and above	3.94	4.16	3.94
**Hypertension History**			
1–5 years	4.43	4.63	4.26
6–10 years	4.23	4.37	4
Over 10 years	4.1	4.25	4.02
**Regular Visit**			
No	4.24	4.42	4.11
Yes	4.18	4.33	4.05
**Regularly Visit Hospital Level**			
Primary	4.13	4.34	3.95
Secondary	4.37	4.35	4.05
Tertiary	4.15	4.31	4.08
**Regular Visit Frequency**			
Every month	4.19	4.3	3.91
Every 3 months	4.12	4.25	4.2
Every year	4.23	4.42	4.02

**Table 7:** One-way analysis of variance of the elderly characteristics to APP quality evaluation

**Characteristic**	**System Quality**			**Information quality**			**Service quality**		
	**Mean difference**	***P*** value	**95% CI**	**Mean difference**	***P*** value	**95% CI**	**Mean difference**	***P*** value	**95% CI**
**Gender**									
Male	0.27^a^	0.042	0.00~0.52	0.14	0.245	-0.10~0.38	0.07	0.503	-0.13~0.27
Female	-	-	-	-	-	-	-	-	-
**Education Level**									
Junior college and below	-	-	-	-	-	-	-	-	-
Undergraduate and above	-0.33^a^	0.026	-0.44~-0.19	-0.25^b^	-0.064	-0.51~0.01	-0.15	0.179	-0.38~0.07
**Hypertension History**									
1-5 years	0.33^a^	0.029	0.03~0.63	0.38^a^	0.005	0.11~0.65	0.24^a^	0.044	0.00~0.47
5-10 years	0.13	0.350	-0.15-0.42	0.12	0.368	-0.14~0.38	-0.02	0.866	-0.24~0.20
Over 10 years	-	-	-	-	-	-	-	-	-
**Regular Visit**									
No	0.07	0.610	-0.19~0.32	0.10	0.403	-0.13~0.33	0.06	0.553	-0.14~0.25
Yes	-	-	-	-	-	-	-	-	-
**Regularly Visit Hospital Level**									
Primary	-0.02	0.928	-0.35~0.32	0.13^a^	0.049	-0.08~0.34	-0.13	0.522	-0.39~0.13
Secondary	0.22	0.271	-0.17~0.61	0.03	0.160	-0.33~0.40	-0.03	0.652	-0.33~0.28
Tertiary	-	-	-	-	-	-	-	-	-
**Regular Visit Frequency**									
Every month	-	-	-	-	-	-	-	-	-
Every 3 months	-0.07	0.698	-0.42~0.28	-0.06	0.719	-0.38~0.26	0.29^a^	0.036	0.02~0.55
Every year	0.04	0.822	-0.30~0.38	0.11	0.479	-0.20~0.43	0.11	0.417	-0.15~0.37

^a^ *P*<.05, ^b^ *P*<.1

## Discussion

### Principal findings

This research aimed to develop and validate a multidimensional, hierarchical scale for assessing the quality factors of mHealth apps used by elderly patients with hypertension in China. A scale with three factors was established based on the DeLone and McLean ISS framework to represent the overall quality of mHealth apps and affected user satisfaction. The three quality factors included were system, information and service quality, constructed with seven independent observational indicators selected from previous studies. The results demonstrated that the scale was valid, and the relationship between the quality factors and users’ satisfaction had empirical support.

The findings of the study indicated that ‘information quality’ and ‘service quality’ have a significant impact on the degree of satisfaction experienced by elderly individuals utilizing hypertension management apps, which was supported by previous literature [47]. ‘Information quality’ was observed to be the most influencing factor on the degree of satisfaction, with timeliness being more important than the other attributes assessed. This may be due to elderly patients valuing timely updates and feedback of information above content relevance and accuracy of health information. Health information acquired through mHealth apps can increase people’s knowledge and personal abilities to further manage their hypertension, which is the most first and essential perception of the quality of mHealth apps. ‘Service quality’ represents the extent to which mHealth services meet patients' needs. According to Akter et al. [48], ‘service quality’ was a higher-order globally construct which includes ‘system quality’, ‘interactive quality’, and ‘information quality’. It contains not only the final service content provided by mHealth, but also the production and delivery process of the service. However, as quality was divided into three parts, ‘service quality’ was a narrow concept, more focused on the service model beyond system quality and information quality. Nonetheless, service quality is an important factor in user’s satisfaction with mHealth [18], as observed in the elderly individuals included in current study. All three proposed indicators had a significant path coefficient on the scale. Among these, ‘interactivity’ was recognized as the most influential indicator of service quality. In China, mHealth has been given an important mission to improve the productivity of healthcare services, to some extent, play an alternative role to some offline services, such as routine monitoring and follow-up. Interactivity, an important feature of face-to-face healthcare services, is an important aspect for patients to perceive the equivalent quality of mHealth services to real-world health services.

Inconsistent with previous research [45], ‘system quality’ did not affect user satisfaction significantly, which is also in contrast to the ISS framework. As an essential prerequisite for information product [28], system quality is often taken for granted and rarely researched in the path analysis of mHealth satisfaction [18]. However, in this study, it was proven to be an integral part of the overall quality of mHealth apps, but not a predictor of user satisfaction, which may reflect the situation of hypertension management apps. Research has revealed that the functions of hypertension management apps are far from meeting patient needs [34]. Owing to the relatively low information literacy of elderly individuals, their understanding of system quality is less profound, therefore, the impact of system quality on the satisfaction of the elderly users is not obvious.

Preliminary results for the quality of mHealth care for elderly individuals were obtained by applying the quality scale in assessing elderly patients with hypertension. The quality results for different factors were uneven. Service quality was a shortcoming of current hypertension management apps. Elderly patients with hypertension are typically characterized by prolonged disease histories and many concomitant diseases, which often require timely communication with physicians and the integration of online and offline services. However, this study showed that the ‘interactivity’ and ‘customization’ quality of current mHealth apps were still not ideal. This problem is reflected in the functionality of system quality and the timeliness of information quality. In addition, previous studies have found the same problem in the perception of patients with COPD regarding mHealth apps [49]. Ji et al. [50] conducted a survey on the use of mHealth apps among 250 middle-aged and elderly individuals in China and found that 46.4% of the participants were not satisfied with the treatment effect. Effective interaction was not only helpful in improving the effect of disease management, but also an important means to meet their spiritual needs, which plays a major role in promoting the use and satisfaction of mHealth apps [51]. The affordability of services was another pitfall that affected the quality of mHealth apps perceived by patients. China has been increasingly promoting that mHealth be covered by the basic medical insurance scheme (BMIS). This process has accelerated since the outbreak of the COVID-19 pandemic. Thirty provinces had already approved subsequent online visits and prescriptions for reimbursement by BMIS. Elderly patients are usually heavily reliant on BMIS to afford their healthcare costs. However, a payment mechanism for chronic disease management is still lacking, which is believed to be an important obstacle for mHealth utilization and promotion. As “Health China 2030” proposed, the healthcare development pattern is transferring from treatment centered to health centered. There is great demand for creative technologies, such as mHealth apps, to enhance the efficiency of chronic disease management. Increasing insurance coverage for such new healthcare modalities should be a necessary option for its promotion to establish a new healthcare pattern. In addition, embedding mHealth into existing health management services, such as the family doctor service and basic public health services, to further improve their service efficiency should also be recommended.

There was significant difference in quality perception among patients of different gender, hypertension histories, and healthcare frequencies. Similar to previous studies, male patients generally accepted mHealth apps more easily [50, 52]. This is because men are generally more receptive to innovations. Elderly individuals who have a long history of hypertension tend to rate the service quality lower. This result is in agreement with many previous studies assessing mHealth apps for chronic diseases [53, 54]. Users have different demands for mHealth at different stages of chronic disease. Due to a relative lack of knowledge about the disease and relatively simple needs for healthcare services in the early stages, mHealth apps could play a major role in monitoring and health management [55]. However, elderly patients with hypertension and a longer disease history often have comorbidities and complications, such as stroke, arrhythmia, which will require more specialized healthcare and examinations beyond the capacity of mHealth. Meanwhile, patients who regularly visited tertiary healthcare facilities gave significantly lower scores for the information quality than those who regularly visited primary hospitals. Existing studies have shown that long-term relationships between physicians and patients have an impact on chronic disease management [55] and evaluation of mHealth apps [36, 56]. In China, the national health administration has made great efforts to allocate family physicians in primary care hospitals to every household in the country, and advocates providing services through mHealth. The signing rate of family physicians among older adults increased from 28.33% in 2015 to 75.46% in 2020. The policy and greater coverage of family physicians strengthens the trust between doctors and patients, which allows efficient communication when needed. However, physicians in tertiary hospitals typically have large workloads which makes it difficult for them to participate in mHealth service for their patients [57]; this leads to users feeling disconnected from their regular healthcare experience when using mHealth. But due to the shortcomings in medicine supply and service capacity of primary care and patients’ healthcare behavior tendency in China, there is still a considerable gap between family physician services and the demands of patients with chronic diseases [58]; this shows that the feature, regular hospital visits to primary hospitals, failed to play a positive role in determining quality perception. In 2019, the National Health Commission of the People’s Republic of China issued a new policy to promote a ‘county-level integrated healthcare organization’ in order for healthcare resources to be optimally allocated across the county to improve community-level health services. MHealth combined with primary healthcare has shown potential effects for patient surveyed; therefore, this new form of health service will play a greater role in promoting continuity of care, thereby providing an improved form of health management for older adults; this is crucial as the problems associated with aging are currently relevant in many countries.

### Theoretical implications

This study provided valuable insight into the evaluation of mHealth apps quality by elderly patients with hypertension. First, this study provides a quality evaluation scale for mHealth apps from the perspective of elderly patients with hypertension, which assists mHealth research by offering a theoretical framework for interpreting the quality expectation of this population. Second, the study proved the hypotheses regarding the impact of quality on user satisfaction. The empirical data supported most hypotheses and had strong explanatory power. Finally, this study contributes to the mHealth service literature by highlighting key patient characteristics influencing the quality evaluation of mHealth, which will enable policymakers and service providers to further understand the cognitive behavior of elderly individuals towards mHealth to reduce their exclusion, especially in developing countries.

### Practical implications

It is important to understand the quality of apps from the perspective of target users to examine the difficulties and issues regarding the development and implementation of mHealth services. This study developed practical tools for evaluating the quality of mHealth apps from the perspective of a high-demand and high-prevalence population, which would aid the planning, designing, and developing of mHealth services for the elderly in China. The findings in this study showed that developers should focus on timely information and interactive services to enhance the satisfaction of elderly individuals with mHealth apps. Besides, this quality perception was different among people with different medical service demands and information literacy, which will assist developers to design different service strategies for targeted groups.

### Limitations

This study has been several limitations. First, due to the sampling method used and the special age groups selected may restrict the generalizability of these findings. However, the demographic and hypertension history of the participants included in this study could be representative of the elderly population with hypertension of China. Further research should extend the evaluation scale to include different patient groups to obtain a more generic view of the theoretical framework. Second, this study is a cross-sectional study, therefore, there may be some unobserved contingent and causality factors influencing patient quality evaluation. Longitudinal follow-up studies using this scale are required to avoid interference of other factors. Finally, this study focused on the quality evaluation of hypertension health management apps for elderly patients; therefore, the classification of apps has not been more detailed. The different mHealth apps operation characteristics and their relationship with patient evaluation are still require further investigation.

## Conclusion

The rapid growth of mobile technology has meant that mHealth has become a promising tool for supporting chronic disease management. It is crucial to understand the perception of older adults regarding their interaction with health management apps to provide a reliable basis for promoting mHealth apps in an ageing society. To enhance the understanding of mHealth quality perception by elderly patients, this study developed and validated a multidimensional, hierarchical scale, and analyzed the impact of the quality factors on user’s satisfaction with mHealth apps in China. The findings suggest that the proposed evaluation scale based on ISS theory has strong reliability and validity. ‘Information quality’ and ‘service quality’ significantly impact elderly patients’ satisfaction with hypertension management apps. However, ‘system quality’ was not found to be significant in explaining satisfaction in this study, which is inconsistent with the existing literature. Nevertheless, this finding is in alignment with the current situation of mHealth development in China, where inadequate system functionality is common. This study showed that developers should consider the different disease-stage characteristics, and closely cooperate with primary care system when designing service models for elderly users to enhance the accessibility of mHealth services. Future research should involve more diverse age groups and longitudinal survey data to thoroughly examine the scale’s validity. More detailed behavior features of older adults and hypertension service factors are intended to explore the mechanisms of different quality levels for hypertension management apps, such as health education and health insurance; this will provide more comprehensive and evidence-based information to support a broader range of behavior change and health outcomes for patients with hypertension.

## Supplementary Information

**Additional file 1.**

## Data Availability

The datasets generated during and analyzed during the current study are not publicly available. However, data will be available from the corresponding author if a specific reasonable request will be transferred.

## Abbreviations

NCD: Chronic non-infectious diseases
WHO: World Health Organization
ANOVA: One-way analysis of variance
