# Supplementary material for: A scale to measure the perceived quality of mHealth by elderly patients with hypertension in China
Source: BMC Health Serv Res. 2023 Apr 10;23:351. doi: 10.1186/s12913-023-09357-z (PMC10088124; doi:10.1186/s12913-023-09357-z)
Supplement: Supplementary file 1 — Additional file 1. [file 12913_2023_9357_MOESM1_ESM.docx]

| **Factors** | **Indicators** | **Items** |
| --- | --- | --- |
| **System** | |  |
|  | Ease of use | The APP is easy to learn |
|  | Functionality | The APP’s functions can satisfy most of my hypertension management activities |
|  | Inter-device  compatibility | The APP can well interact to my other hypertension management devices well |
|  | Ease of navigation | The various functions in the APP were well integrated and navigated to be used |
| **Information** | |  |
|  | Relevance | The information provided by this APP is very relevant |
|  | Accuracy | The information in this APP is scientific based on my knowledge |
|  | Timeliness | The hypertension knowledge and advices from physicians is updated timely |
| **Service** | |  |
|  | Interactivity | I can effectively communicate with online physicians through this APP |
|  | Customization | This APP can be well integrated with my daily hypertension treatment activities |
|  | Affordability | The charge for the service provided by this APP is reasonable |

**Appendix A: Questionnaire Items for Quality Evaluation Hypertension Management APPs**
